# Outcomes of Guided Personalized Surgery (GPS)-Navigated Reverse Polarity Total Shoulder Joint Replacement in a Low-Volume Hospital

**DOI:** 10.7759/cureus.50622

**Published:** 2023-12-16

**Authors:** Narada R Karuna Pathirannehelage, Jithuram Jayaram, Indika S Bamunuarachchi, Joby J George Malal

**Affiliations:** 1 Trauma and Orthopaedics, Bedfordshire Hospitals NHS Trust, Bedford, GBR

**Keywords:** reverse polarity total shoulder joint replacement, reverse shoulder arthroplasty, gps-navigated rsa, glenoid version and bone loss, oxford shoulder score, glenoid base plate placement, guided personalised surgery

## Abstract

Introduction

Reverse polarity shoulder arthroplasty (RSA) is an evolving surgery, and its indications have expanded over time. Apart from cuff tear arthropathy (CTA), it is recommended for complex proximal humerus fractures in the elderly, inflammatory arthritis, primary osteoarthritis in the elderly, and revision for failed hemiarthroplasty. Glenoid base plate placement and fixation are important to prevent complications, especially glenoid base plate loosening, dislocation, and scapular notching, and to improve longevity. Guided personalized surgery (GPS)-navigated RSA was devised to optimize the glenoid base plate position and fixation.

Methodology

A retrospective study was carried out in a low-volume district general hospital in England. All the patients who underwent GPS-navigated RSA were included. Their preoperative glenoid version, bone stock, glenoid base plate, and glenoid screw lengths were analysed. Preoperative and post-surgery patient-reported outcomes were gathered using the Oxford Shoulder Score (OSS) at six months and annually thereafter.

Results

Fourteen patients have undergone GPS-navigated RSA in our institute since 2018. Ten patients were female. All of them had a retroverted glenoid with a mean value of 13.6 degrees. Ten out of 14 patients had an augmented glenoid base plate. This included six eight-degree posterior augmentations, three 10-degree superior augmentations, and one extended cage peg. The follow-up period was six months to five years, depending on the date of surgery, and none of the patients dropped out of follow-up. The OSS revealed statistically significant improvement from preoperative values to six months postoperative, an improvement of 21.64±7.175. It also showed progressive improvement over time during postoperative follow-up, and the three-year mean was 47. The commonest complication was fractures, which happened in four cases. There were no infections or dislocations.

Discussion

Guided personalized surgery-navigated RSA was performed on selected patients at our institution when they were not suitable for conventional RSA due to distorted glenoid anatomy. Glenoid base plate positioning and fixation are important to optimize the outcome of RSA. Guided personalized surgery navigation is helpful in achieving optimum glenoid base placement, especially when the normal glenoid anatomy is distorted. There were no dislocations, glenoid base plate loosening, or scapular notching in the study group. There were four reported fractures, which was comparable with the published literature.

## Introduction

Shoulder joint arthroplasty has been evolving for the last two to three decades. Anatomical total shoulder arthroplasty (TSA), resurfacing arthroplasty, and hemiarthroplasty dominated shoulder arthroplasty in the early decades. Reverse polarity total shoulder arthroplasty (RSA) has become more popular over the last decade [1].

A TSA needs a functional rotator cuff according to its design and biomechanics. One of the most common complications associated with TSA is humeral head subluxation, which is directly related to cuff tears [2, 3]. It is very common to have cuff tears in the elderly population. The incidence is more than 50% at the age of 80 [4]. Therefore, TSA becomes a less viable option in the elderly population, and complication rates increase as patients get older. Reverse polarity total shoulder arthroplasty was introduced as a replacement option for cuff-deficient shoulders. It moves the centre of rotation of the shoulder medially and inferiorly. This creates an increased lever arm for the deltoid muscle, allowing it to act as a prime shoulder abductor. Glenoid base plate positioning is important in RSA. Poor placement leads to glenoid base plate loosening, which is one of the most common complications of RSA [5, 6]. Improper positioning of the central peg and screws contributes to early loosening. Further screws can penetrate the cortex and increase the risk of neurovascular injury.

Good glenoid base plate placement and fixation require good bone stock and anatomical landmarks of the glenoid. In the presence of advanced osteoarthritis or cuff tear arthropathy (CTA), both the bone stock and the glenoid landmarks are distorted. Glenoids become retroverted with significant bone loss. Sometimes bone cysts significantly compromise the bone. In these situations, conventional RSA becomes a challenging operation, and the outcome is compromised due to improper glenoid base plate positioning and fixation.

Guided personalized surgery (GPS)-navigated RSA helps achieve better glenoid base plate positioning and fixation in these conditions [7, 8]. The process involves a three-dimensional (3D) CT of the shoulder and a preoperative planning application to create GPS navigation based on the CT images. During the operation, the trackers are used to guide the position of the base plate and screws.

There is evidence in the literature that suggests GPS-navigated RSA helps achieve good position of the glenoid base plate and secure peg and screw fixation [7, 8]. We did a retrospective case series study to analyse the demographic data, implants, and functional results of GPS-navigated RSA at our institution.

## Materials and methods

A retrospective descriptive case series study was carried out at the Bedfordshire Hospitals NHS Trust, Bedford, UK. The study was registered in the Clinical Quality, Audit, and Effectiveness Department of the Bedfordshire Hospitals NHS Trust. The main objectives were to assess functional improvement after a GPS-navigated RSA, establish commonly used glenoid base plates and screws, and define the accuracy of the glenoid base plate and screws' placement. Our hospital is a low-volume district general hospital in England, where navigation was used only for challenging cases. In 2018, five years ago, our institute began using GPS-navigated RSA. All the patients who were planned for GPS-navigated RSA during the last five years were included. Patients who did not complete GPS-navigated RSA for any reason were excluded from the postoperative functional assessment. Preoperative modified anteroposterior (AP) and axillary lateral radiographs were analyzed for proximal migration and arthritic changes.

Further, axial views of CT scans were used to measure the glenoid version and assess for bone stock. Intraoperative glenoid base plate placement under navigation was checked to assess the accuracy of the placement in comparison to the preoperative plan. Postoperative AP and axillary lateral views were analysed to assess the position of the peg and screws. The length of the screws used to fix the glenoid base plate was also studied.

The Oxford Shoulder Score (OSS) was calculated from the patient-reported outcome measure form before the operation and at six months, one year, and then yearly at postoperative follow-up.

Surgical technique

The deltopectoral approach was used in all patients who were positioned on beach chairs. A GPS screen was fixed to the opposite side of the table without any obstructions so that trackers could be easily sensed. A static tracker was positioned on the coracoid, and the glenoid anatomy was acquired using a tracked pointer. After the completion of the glenoid mapping, glenoid reaming was done under navigation guidance with a tracker affixed to the reamer handle, giving real-time feedback on the angle and depth of reaming. The central screw and glenoid base plate were positioned using tracked instruments. All the base plates were fixed with four screws inserted under navigation for maximum purchase and optimum positioning. The rest of the procedure was like a standard RSA.

IBM SPSS software version 26 (IBM Corp., Armonk, NY) was used for statistical analysis. Descriptive statistics were mainly used, and a paired t-test was used to compare the OSS before and six months after the operation.

## Results

Fifteen patients were planned for GPS-navigated RSA at our institution over the last five years. There was one case of attempted navigational RSA that was converted to a standard RSA due to a coracoid fracture when the coracoid tracker was being fixed. All the surgeries were performed by a single surgeon. Equinoxe Exactech (Exactech, Inc., Redditch, UK) implants were used in all the patients. Apart from the complication analysis, the patient who did not complete GPS-navigated RSA was excluded from the study.

Ten female patients and four male patients were included in the study. The average age of the patients was 71.3 years, with a range of 57 years to 88 years. 

Two patients had CTA with proximal humerus migration and complete loss of subacromial space. The other 12 patients had primary osteoarthritis without radiological evidence of a cuff tear. A clinical decision was made to proceed with GPS-navigated RSA because of significant glenoid bone loss or retroversion. No single cut-off value for the retroversion was used during decision-making. The senior author, who was the primary surgeon, made a clinical decision considering all the factors.

All the patients had retroverted glenoids ranging from two degrees to 35 degrees. The mean version was 13.6 degrees. Four patients had a retroversion of more than 20 degrees with severe posterior bone loss. One patient had severe central bone loss. Four patients had large bone cysts in the glenoid, compromising more than 30% of the bone stock.

**Figure 1:**
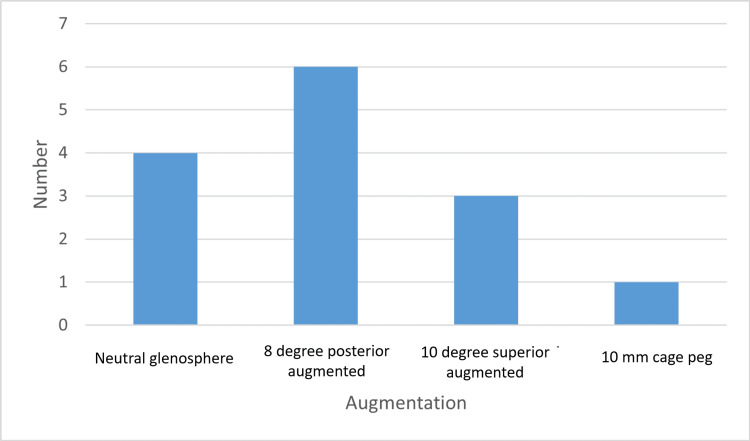
Glenoid base plate augmentation

Five patients had eight-degree posteriorly augmented glenoid base plates. Three patients had 10-degree superiorly augmented glenoid base plates (Figure 1).

In 10 of the 14 patients, the most commonly used glenosphere size was 38 mm. Four patients had 42-mm glenospheres.

The glenoid version was accurately reproduced intraoperatively according to the preoperative plan in all patients. The range of screw lengths was 24 mm to 37 mm. The mean screw length was 28 mm. All patients had four screws to fix the glenoid base plate.

The follow-up period was from six months to five years, depending on the date of the surgery. None of the patients were dropped out of follow-up. The OSSs of the patients are depicted in Table 1.

**Table 1:** The Oxford Shoulder Scores of the patients

	Preoperation	6 months	1 year	2 years	3 years	4 years	5 years
Count	14	13	12	8	3	1	1
Mean	17	38.9	42	44.9	47	48	36
Mode		38	42	48			
Median	18	40	42	45	47	48	36
Range	23	16	16	10	2	0	
Minimum	1	30	32	38	46	48	36
Maximum	24	46	48	48	48	48	36
Standard deviation	5.9	4.4	4.9	3.3			
Standard error	1.6	1.2	1.42	1.17	0.58	0	0

A paired sample t-test was used to assess the improvement in OSS over the first six months compared to the preoperative OSS recorded on the day of surgery (Table 2).

**Table 2:** Paired t-test results OSS: Oxford Shoulder Score

Paired sample t-test									
		Paired differences					t	df	Sig. (two-tailed)
		Mean	Standard deviation	Standard error mean	95% confidence interval of the difference				
					Lower	Upper			
Pair 1	Preoperative OSS - Six-month postoperative OSS score	-21.643	7.175	1.918	-25.785	-17.500	-11.287	13	.000

There was no glenoid loosening or dislocations during the follow-up period. One patient developed a periprosthetic humerus fracture at the tip of the stem, which was managed with osteosynthesis. One patient developed a coracoid fracture one month postoperatively, which was managed non-operatively. One stress fracture of the acromion was reported, which was managed non-operatively as well.

## Discussion

Shoulder arthroplasty has evolved over the last four decades. Reverse shoulder arthroplasty is becoming more popular among shoulder surgeons worldwide. Indications have expanded over time. A definite indication is arthropathy of the shoulder in the absence of a functioning rotator cuff. Reverse shoulder arthroplasty is also preferred in inflammatory arthritis, non-reconstructable proximal humerus fractures in the elderly, primary osteoarthritis in the elderly population, and revision for failed hemiarthroplasty [9-13]. Reverse shoulder arthroplasty is preferred over TSA in the elderly population due to the high incidence of rotator cuff failure and proximal humeral migration.

Reverse shoulder arthroplasty is not immune to complications. Glenoid base plate loosening is one of the most common complications [9, 14]. It is crucial to get the glenoid base plate positioning and fixation optimized to improve the longevity of RSA [15]. Most of the time, careful exposure and adherence to the anatomical landmarks will lead to good glenoid base plate positioning. The usual technique is a guidewire inserted at the centre of the glenoid perpendicular to the glenoid. Reaming is done with cannulated reamers, which pass over the wire. When the normal anatomy is significantly distorted, achieving the correct glenoid base plate becomes difficult. It is further complicated by the perforation of the cortex by the screws, leading to poor fixation or neurovascular injuries. In addition to the distorted anatomy, poor glenoid bone stock also compromises glenoid fixation. This can be due to large glenoid cysts or glenoid bone erosion.

Navigated RSA is useful in these situations to optimise the glenoid base plate positioning and fixation. A preoperative CT scan is done, including 3D reconstruction. This information is used to prepare a 3D model of the scapula and preoperative planning, which is then used to guide implant positioning. During the surgery, with the guidance of trackers and pointers, implant positioning is established as planned by the software.

In our institution, GPS-navigated RSA was introduced in 2018. We use the Equinoxe Exactech shoulder system. Patients who were not suitable for conventional RSA were selected for GPS-navigated RSA. This helped to improve our service, and we were able to perform the procedure on patients who were not suitable for a conventional RSA.

Fifteen selected patients were planned for GPS-navigated RSA out of a total of 156 RSA. Fourteen patients underwent GPS-navigated RSA, and one patient was converted to conventional RSA due to a coracoid fracture. A GPS-navigated surgery was not offered to everyone as it was not considered cost-effective for routine cases. All of the patients who underwent GPS-navigated RSA had distorted retroverted glenoids. During the operation, 10 out of 14 patients required an augmented glenoid base plate. All of our patients had four screws, and the average length of the screw was 28 mm. In comparison to the cadaveric study on GPS-navigated RSA, our average screw length was shorter [8]. This could be due to distorted anatomy in our patient group compared to normal cadaveric shoulders.

The longest follow-up for our patient population was five years. The OSS increased to 38.86±4.22 in six months. This is an improvement of 21.64±7.175 compared to the preoperative value of 17.21±5.90. This is statistically significant, with a t-value of -11.287 and a p-value <0.05. All of the cases showed gradual improvement in OSS during the follow-up, except for the patient who had a periprosthetic humerus fracture at four years postoperative.

Eng et al. did a systematic review of GPS-navigated TSA vs. conventional TSA [7]. We did not do a comparison study as our patients who underwent GPS-navigated RSA were more complex cases and hence not comparable to the non-GPS cohort. All of them had glenoid retroversion and distorted glenoid morphology, which would hinder or complicate conventional RSA. Eng et al. systematic review mostly describe the accuracy of the restoration of the glenoid version using navigation. Our patients did not have postoperative CT scans, and it was difficult to assess their version accurately based on radiographs. We accurately reproduced the version intraoperatively according to the preoperative plan for all 14 patients. Our outcome is comparable to the published literature for conventional RSA from the perspective of patient-reported outcome measures and incidence of complications [9, 10].

The most common complication in our study population was fractures. This is comparable to reports in the literature [9, 10]. This includes one postoperative and one intraoperative coracoid fracture. One acromion stress fracture and a humerus shaft periprosthetic fracture following a fall. All the fractures were in females. A small and osteoporotic coracoid may be a contributing factor. As the coracoid tracker is essential for the operation, it is crucial to be careful when fixing the coracoid tracker. We had to abandon GPS navigation for one patient due to a coracoid fracture. Acromion fractures are stress fractures due to increased activity of the deltoid after RSA. Gradual rehabilitation and muscle strengthening are important for these patients. There were no other significant complications in our group of patients. There were no infections or dislocations in our group of patients, which is contrary to the published incidence [9, 10]. The smaller number of patients in our study may explain the differences in the complications profile.

There are a number of limitations to the study. Being a low-volume centre, our study population is small. Our study is a non-randomised descriptive study. A randomised study to compare the outcome of navigated RSA to conventional RSA is recommended to further prove the effectiveness of navigated RSA. Our patients did not have postoperative CT scans; hence, the postoperative glenoid base plate version was not measured. The maximum follow-up duration was limited to five years. Longer follow-up is needed to further establish the long-term outcome.

## Conclusions

Guided personalized surgery-navigated RSA shows good short-term results in patients with distorted glenoid anatomy where conventional RSA would be challenging. It gives a good functional outcome within six months and gradual improvement thereafter. While it is essential to investigate the long-term results more thoroughly, our findings recommend the use of GPS-navigated RSA in selected patients with distorted glenoid anatomy. Further research work, including a comprehensive and representative sample, is recommended.
